# Farmers’ Training on Pesticide Use Is Associated with Elevated Safety Behavior

**DOI:** 10.3390/toxics5030019

**Published:** 2017-08-22

**Authors:** Christos A. Damalas, Spyridon D. Koutroubas

**Affiliations:** Department of Agricultural Development, Democritus University of Thrace, GR-682 00 Orestiada, Greece; skoutrou@agro.duth.gr

**Keywords:** farmers, worker protection standards, safety behavior

## Abstract

Occupational exposure to pesticides in agricultural applications may cause acute and long-term health effects to farmers, and thus research on factors that reduce exposure is useful. However, studies on the relevance and effectiveness of training are limited. The association of previous training in the form of intensive seminars relating to pesticide use (e.g., use of spraying equipment, application parameters, use of personal protective equipment, risks to human health and the environment) with farmers’ knowledge and behavior in pesticide use was studied via the self-reporting method in a purposive sample of 82 trained and non-trained farmers. Most trained farmers showed higher levels of knowledge of pesticide use, higher levels of beliefs in pesticide hazard control, and higher levels of safety behavior than non-trained farmers. Knowledge of pesticide use and beliefs regarding pesticide hazard control were significantly correlated with safety behavior in both groups of farmers. Concerning farmers’ beliefs regarding pesticide hazard control, trained farmers were more likely to think that safety precautions work very well and less likely to feel they had little control over avoiding pesticide hazards. Overall, previous training was associated with increased levels of farmers’ knowledge of pesticides and beliefs about pesticide hazard control, was accompanied by elevated safety behavior in farmers, and thus was connected with lower occupational exposure to pesticides. Interventions that facilitate knowledge and compliance with safety behaviors should become a priority for decreasing exposure to pesticides among farmers.

## 1. Introduction

The use crop protection products has boosted the productivity of land, reduced the need to farm additional land, and contributed to greater and more stable income for farmers [1]. Pesticides protect crops from harmful pests and diseases, assisting farmers to secure food supply with an efficient use of natural resources. Farmers rely on pesticides, including toxic chemicals, to a greater degree than traditional pest control methods (e.g., physical and mechanical control) and integrated pest management (IPM) [2]. Due to their ease of use and high efficacy, the use of pesticides has increased over the years [3]. However, misuse of pesticides occurs, particularly in developing countries, generating serious concerns of personal and environmental safety [4]. The consequences of the misuse of pesticides lead to ecological imbalance and environmental pollution. In addition, over-reliance of farmers on pesticides, lack of knowledge of proper handling practices, and poor access to training on pesticides imply a high risk of pesticide exposure for farmers and pesticide residues on crops [5]. The high level of risk exposure to pesticides among farmers calls for immediate intervention aimed at increasing awareness about alternative pest control practices with lower pesticide use [6]. The use of the appropriate type of spraying equipment, the use of proper protective clothing when handling pesticides, and the adherence to correct spraying practices have been found to be critical factors influencing the degree of pesticide exposure among those applying pesticides [4,5,6].

The use of pesticides will always involve some degree of risk, because of the poisonous character of these chemicals [7]. Farmers and their family members run the highest risks, as they can easily come into contact with the pesticides when mixing the chemicals or when applying them to the crop. Hundreds of cases of poisoning in the developing world, where information and training on the potential negative health effects of these chemicals is often lacking, are attributed to pesticides. Acute poisoning with pesticides is a global public health problem, accounting for as many as 300,000 deaths worldwide every year [8]. Many of these pesticide poisonings, particularly in the developing world, are intentional [9]. A conservative estimate by Mew et al. [9] reported approximately 110,000 pesticide self-poisoning deaths each year from 2010 to 2014, comprising 13.7% of all global suicides. Apart from target organisms, other organisms (e.g., beneficial insects, birds, earthworms, and fish) can be affected by pesticides in or around fields, resulting in death of wildlife, death of farm animals, and loss of biodiversity [10]. Moreover, the destruction of beneficial insects that often occurs interferes with natural pest control, a fact that can cause new pest problems [11]. This means that minor pests, which are usually kept at low populations by their natural enemies, could multiply rapidly in the absence of their enemies and cause outbreaks. In this case, the control directed against one pest may result in the outbreak of another pest. Another problem of indiscriminate pesticide use is that pests can become resistant to the chemicals. Without guidance for dealing with this issue, farmers often decide to spray more frequently and to apply high pesticide doses. This just causes more problems, such as increased exposure to pesticides and high chances of additional pesticide resistance. In addition, pesticides that are sprayed on the crop can leave behind residues that can be eaten by consumers, with differing exposure cases between populations in different countries of the world [12].

Training is likely to be an important intervention for reducing farmers’ exposure to pesticides [13]. Safety training is defined as ‘instruction in hazard recognition and control measures, learning safe work practices and proper use of personal protective equipment, and acquiring knowledge of emergency procedures and preventive actions’ [14]. Training of smallholder farmers on IPM and good agricultural practices in farmers’ field schools (FFS) in Bolivia had positive effects (e.g., improvement on the use of PPE and hygiene when handling pesticides, knowledge and use of IPM and ecological alternatives of pest control, and a reduction in self-reported symptoms after pesticide handling), but is scarce in most low-income countries [15]. However, differences in the perceived importance and competence of farmers on the safety measures revealed considerably different needs of farmers for future training as a result of differences in age along with other background characteristics [16]. Poor uptake of training by farmers on pesticides and the aging farming workforce are causes for concern. Studies on the relevance and effectiveness of training are limited. Thus, evaluation of training by any available means, as a systematic process of gaining insight into a training activity and its effects, can be used for guiding decision-making and for designing more effective training components. In this context, the objective of the study is to assess the effectiveness of previous training in improving farmers’ knowledge and attitudes in pesticide use.

## 2. Methodology

### 2.1. Study Area and Sample Selection

The study was carried out in a purposive sample of 82 trained and non-trained farmers from rural areas of northern Pieria (Eginio and Methoni) in northern Greece. The survey consisted of interviews with farmers from areas where mainly cotton is cultivated, along with other crops. The farmers were purposively selected from a totally random sample of cotton farmers of the above regions on the basis of previous training on pesticide use, including use of spraying equipment, application parameters, use of personal protective equipment, and risks to human health and the environment. Thus, to better serve the objective of the study, we chose to compare two groups of farmers, i.e., the trained group, consisting of farmers who had received training in the past, and the non-trained group, consisting of farmers who had never received training. Therefore, training was the only inclusion criterion for the trained group. Neither group was stratified by age, education, or any other demographic variable. Previous training was in the form of an organized seminar with classroom lectures or field demonstrations or both that required registration of participants. Training was provided by research units of agricultural institutes or by major agrochemical companies. Identification of the exact time of previous training was not feasible for most farmers.

Purposive sampling, also known as subjective sampling, is a non-probability sampling technique where the prospective participants of the survey are selected based on the judgment of the researcher [17]. A purposive sampling aims at focusing on particular characteristics of a population that are of interest for the study, a fact that allows reaching appropriate participants for the research questions set. In this context, the sample was not representative of the population, but this issue is not considered to be a weakness, because the primary consideration in this kind of sample is the judgment of the researcher as to who can provide the best information to achieve the objectives of the study with significant saving in time and money, and not to represent the whole population [18]. Furthermore, purposive sampling is helpful for pilot studies and for hypothesis generation. Therefore, the sample of the study consisted of non-trained farmers and trained farmers with regard to pesticide use, with the aim of gaining insights into differences in safety behavior between farmers who had received training and those who have not.

Potential participants were approached independently considering their availability and their willingness to participate in the study. Farmers were enlisted from lists obtained from the local farm supplies stores in each studied area. The farmers gave oral consent to participate in the study after hearing a brief explanation of the study’s objective. To avoid any potential bias, it was made clear to the farmers that the study was for academic research. In total, 82 interviews were included in this study. The study was submitted for review by an ethics committee, but ultimately did not require approval because it was a simple observational evaluation exclusively for academic research purposes with full anonymity of participants.

### 2.2. Data Collection

A questionnaire with structured items was designed based on previous literature on relative subjects [19]. Although a great variety of items could have been included in the questionnaire, the items were selected on the basis of simplicity, to facilitate understanding by farmers, and of originality in terms of frequency of use in previous publications. Therefore, we tried to select original items to increase the novelty of our study and we kept the suggested answers simple, so that they could be easily understood by farmers, irrespective of education level. The selected items reflect issues of knowledge, beliefs, and behaviors closely related to exposure during pesticide application. The questionnaire consisted of four sections. Section 1 included questions relating to age, education, farm size under cultivation, membership in local associations, previous training on pesticide use (as described above), perception of pesticide hazard, and any episodes of pesticide intoxication in the past (if a farmer felt any kind of sickness from the use of pesticides). Section 2 included questions about farmers’ knowledge of pesticide use, which was assessed with closed-ended questions (rated with 0 = wrong and 1 = correct). Section 3 included questions about beliefs and perceptions of risk, which were assessed with closed-ended questions (rated from 0 to 3). Section 4 included questions about safety behavior during pesticide use, which were assessed with closed-ended questions (rated with 0 = never, 1 = sometimes, and 2 = always). Data were collected through face-to-face interviews with the farmers by means of a friendly discussion. The questionnaire was initially pre-tested using small samples of farmers in the same areas in the form of a pilot study. Data were summarized in three main areas (pertaining to knowledge, beliefs, and behaviors), according to the sections of the questionnaire.

### 2.3. Data Analysis

Descriptive statistics (relative frequencies and means) were calculated for each examined variable. Cross-tabulation was used to summarize the most important categories. The independent samples *t*-test, the Mann-Whitney U test, and the Chi-square test were performed to compare variables between trained and non-trained farmers. Knowledge, beliefs, and behaviors of farmers were classified into frequency (contingency) tables with two attributes (trained and non-trained farmers). This classification was used to determine whether the distributions of trained and non-trained farmers in each variable are the same or different. The Chi-square test for unpaired data was used to determine significant differences between the two independent attributes. However, this test has certain limitations, i.e., no category may have an expected frequency of less than five. The overall score of knowledge, beliefs, and behaviors of farmers was calculated and compared between trained and non-trained farmers. Correlation between variables was determined by Pearson’s correlation coefficient analysis. The effect size was calculated as the mean difference over the pooled standard deviation to examine the magnitude of significant differences [20].

## 3. Results

Basic features of the study population are presented in Table 1. The mean age of trained farmers was 31.14 years, which was lower than that of the non-trained farmers (42.72 years). Most trained farmers had higher education levels than non-trained farmers. Farm size and the proportions of farmers’ membership in associations did not differ significantly between the two groups. Non-trained farmers did not differ significantly with trained farmers with respect to an episode of pesticide intoxication in the past, nor in terms of perception of pesticide hazard, albeit the lower levels of perception of pesticide hazard among non-trained farmers. 

The knowledge levels of farmers about issues of pesticide use are summarized in Table 2. Most trained farmers showed higher levels of knowledge of pesticide use than non-trained farmers, except for the item about the property of a pesticide that would make it more likely to move with water in surface runoff, for which no significant differences between the two groups were found. Disposing of pesticide containers was correctly identified by most farmers of the study, whereas the property of a pesticide that would make it more likely to move with water in surface runoff was correctly identified by the fewest farmers.

Table 3 summarizes farmers’ beliefs about pesticide hazard control. Most trained farmers (80.6%) felt that pesticides can harm health enough to worry a great deal, but this was not the case for non-trained farmers. Trained farmers were more likely to think that safety precautions work very well and less likely to feel they had little control of avoiding pesticide hazards.

Common behaviors of farmers with respect to safety are summarized in Table 4. Most trained farmers showed higher levels of safety behavior in pesticide use than non-trained farmers, except for the item related to washing hands after pesticide application, for which no significant difference between the two groups was found. Washing hands and taking a shower after pesticide application were reported by most farmers, whereas wearing gloves in the preparation of the spraying solution was reported by the fewest farmers.

Summarizing, trained farmers showed significantly higher levels of knowledge, beliefs about pesticide hazard control, and safety behaviors in pesticide use than non-trained farmers (Table 5). Previous research found an increment in perceived risk associated with driving following advanced driving training, showing that training that enhances perceived risks can be designed [21]. The same finding is also applicable to the current study. Knowledge and belief scores were significantly correlated with the safety behavior score, indicating a mediating effect on safety behavior. Farmers have different levels of knowledge and perspectives on risk and the potential negative resulting outcomes from exposure to them. 

## 4. Discussion

The present study explores differences in knowledge of pesticide use, beliefs about pesticide hazard control, and safety behaviors among farmers who have received training and those who have not, using a purposive sample of 82 farmers. It was found that previous training was associated with increased levels of farmers’ knowledge of pesticides and beliefs about pesticide hazard control, was accompanied by elevated safety behavior in farmers, and thus was connected with lower occupational exposure to pesticides. Training was provided by research units of agricultural institutes or by major agrochemical companies in the form of intensive seminars. Knowledge and belief scores were significantly correlated with the safety behavior score, indicating a mediating effect on safety behavior.

The results provide strong empirical support for the hypothesis that knowledge of pesticides and beliefs related to pesticide hazard control are closely associated with safety performance. They generally confirm the conclusions of numerous previous studies [13,15,22,23,24,25] on the positive association of training with farmers’ attitudes to pesticide use. The novelty of the current study lies in the fact that, using original items (in terms of frequency of use in previous publications), it examines together and relates knowledge, beliefs, safety behavior in pesticide use, suggesting that both knowledge and beliefs are important components in shaping safety behaviors. Evaluation of training by any available means as a systematic process of gaining insight into the effectiveness of a training activity can be used for guiding decision making and for designing more effective training components. In this context, the present study provides additional evidence concerning training on pesticide use and its association with three levels that compose safety, i.e., farmers’ knowledge, beliefs, and behaviors. Moreover, the findings provide valuable guidance for researchers and practitioners for identifying mechanisms by which they can improve the safety of the workplace. For example, beliefs involving the impact of risk on the farmers’ safety and the perceived benefit of risk taking should be a factor that is assessed when considering or evaluating the motivation underlying the resulting behavior. From this point of view, this information could be used in the design of interventions with emphasis on all mediating components, namely knowledge and beliefs, to produce the best results.

The most important finding of this study is the positive role of safety training in safety management. It was found that knowledge of pesticides and beliefs about pesticide hazard control were associated with safety compliance. This association could also be attributed, to some extent, to the younger age and the higher levels of education of the trained farmers of this study, which likely contributed to the elevated knowledge levels of those farmers compared with their non-trained counterparts, but this relationship was not tested. On the basis of the results of this study, first it is imperative to give the highest level of priority to safety training convincing the farmers about the need for safety performance. Safety training may be designed to impart good knowledge about the various processes, associated hazards and the safety measures to be taken by the employees in case of emergencies, and improve erroneous beliefs that may exist among farmers. Second, such safety training programs may be conducted regularly and the participation may be made compulsory. To motivate the farmers, they should be given opportunities to actively participate in discussions. However, it is not enough to introduce only the theoretical components of safety behavior in such programs. Rather, both theoretical and farm practice aspects of safety measures, such as the use of personal protective equipment (PPE), should be incorporated for the promotion of farmers’ safety behavior. Very often, education provided to rural workers is barely useful, even when it is not merely ‘symbolic’ and only performed in the first place to comply with European Union (EU) laws, rather than to provide real education to workers. Third, regular evaluation of safety knowledge, level of safety motivation, and safety skills also must be made an integral part of safety training programs. The study also demonstrated that perceptions of safety management practices can influence safety performance.

Safety training is a major strategy for risk prevention to guarantee every employee is safe in good workplace conditions [14]. Safety training has been recognized as an important organizational characteristic, distinguishing organization with successful safety programs, and is an effective means for employees to enhance their skills and knowledge of safety in the organizations [26]. Besides, there is a legal requirement across EU Member States to establish National Action Plans (Directive 2009/128/EC), thereby setting quantitative objectives, targets, measures, timetables and indicators to reduce risks and impacts of PPE use on human health and the environment. In this context, sufficient training would ensure that pesticide users, distributors, and advisors acquire sufficient knowledge of how to identify and control hazards and risks to humans associated with pesticides [19,27].

Farmers’ knowledge of pesticides and safety behaviors in daily practice is important for providing valuable information that can contribute to educational and policy recommendations aimed at preventing or reducing health and environmental hazards associated with pesticides. It has been reported that poor knowledge regarding pesticide risks and handling among inhabitants of the Vehari District in Pakistan contributed to high exposure levels to organochlorine pesticides, particularly among farm workers [22]. As all stages in the use of pesticides on farms could generate risks for the operator and the environment, a basic recommendation is to promote training for pesticide users and adoption of good agronomic practices to improve sustainable use of pesticides [28]. To achieve sustainable use of pesticides, it is necessary that everyone is conscious about the risks to both human health and the environment associated with the use of plant protection products. Therefore, raising awareness and training of all relevant stakeholders plays a key role in achieving the objectives of the directive [29]. However, it should be kept in mind that training farmers alone may not be sufficient to reduce pesticide risk. Other strategies should also be employed to complement interventions that provide farmers with information and knowledge of pesticide safety. Enforcing existing pesticide laws and regulations should receive greater attention through surveillance and monitoring activities. For example, there should be a pesticide safety certification program for pesticide applicators and retailers to ensure that only those certified are allowed to sell, handle, or apply pesticides. From a regulatory perspective, addressing issues of pesticide regulation by identifying the jurisdictions that are struggling with these issues, identifying the pesticides that are most often being regulated, and identifying the span and distributions of the values being applied to these pesticides, are other efforts that could help human health risk control [30]. Retention of knowledge is also essential. Retention could be achieved by socializing with farmers whenever possible, so that training messages are communicated through daily routines and spread through the community, increasing the numbers reached. Moreover, farmer-to-farmer training allows farmers to gain an even deeper understanding of the subject matter by becoming educators themselves. A recent study concluded that pesticide protective behaviors in the field may be improved by utilizing moderately experienced farm workers as lay advisors to reinforce training [31].

As in every empirical study, the findings of this study are not without limitations. This project relied mainly on self-reports of farmers’ PPE use and these behaviors were only partly validated against actual use. This might be considered to be an inherent limitation of this study. Self-report studies may suffer limitations of this kind, as people frequently want to report socially desirable behaviors. Thus, validation of answers with the actual behavior of farmers was attempted by visiting some fields with spraying activities without warning and by contacting the local farm supplies stores to collecting extra information. Since no significant deviation was observed between the actual and self-reported behaviors, it was assumed that the possibility of misreporting was negligible (i.e., did not affect the general trends of the study). Moreover, the interviews in this study were conducted in a friendly way and there was good cooperation from the growers, without any refusals, which enhanced confidence and sincerity. Secondly, this study adopted a cross-sectional research design. Hence, no causal inferences could be made for the population; such a statement of causal inferences requires collection of longitudinal data. Therefore, future studies should use a longitudinal design to detect variations over time. Thirdly, the study design does not allow us to know whether differences observed in trained and untrained farmers exist because of the training or because of some other variable. Future studies could also integrate other components of safety to obtain a better view of farmers’ safety behavior.

## 5. Conclusions

Occupational pesticide exposure is common among farmers. The present study showed that previous training was associated with increased farmers’ knowledge of pesticides and beliefs regarding pesticide hazard control, was accompanied by elevated safety behavior, and thus was connected with lower occupational exposure to pesticides. Therefore, interventions that facilitate knowledge and compliance with safety behaviors can be effective in decreasing farmers’ exposure to pesticides, and should become a priority. Promoting the development and facilitation of lifelong learning related to pesticide use should be a priority for minimizing risks to human health and the environment. It should be kept in mind that the problem is not whether a farmer receives training or not, but whether he receives the right training. Therefore, the levels of literacy among farmers and appropriate communication schemes should be considered for a wide range of recipients. Findings of this study may enable regulatory agencies to make better-informed decisions and policy recommendations for reducing potential hazards associated with pesticides. Among policy measures of the EU, National Action Plans (NAPs) that (i) group together the measures set up to implement EU legislation pertaining to pesticides and (ii) set up individual objectives with measures and timetables to achieve them are implemented. The NAPs include important issues (among others), such as creation of a system of awareness-raising and training of professional pesticide users, distributors and advisers, compulsory inspection of the application equipment, enhanced protection of the aquatic environment, and implementation of principles of Integrated Pest Management (IPM) by professional pesticide users. Disparities in levels of knowledge identified in this study could also be used to design training programs with tests of knowledge for farmers. Such training programs on pesticide safety and on the hazards of pesticide exposure should be developed to address gaps in farmers’ knowledge about pesticides (e.g., pesticide drift and run-off). In this context, the national authorities should play a pivotal role by providing up-to-date, accurate, and easy to understand information in the training of farmers to inspire confidence and trust among farmers. Farmers’ sources of information and the degree to which they trust the informants may shape their perceptions of pesticide risk and the adoption of preventative measures.

## Figures and Tables

**Table 1:** Basic characteristics of the farmers surveyed.

Variable	Trained	Non-Trained	Statistic	Value	*p*	Effect Size
Age (years)	31.14	42.72	*t*-test	3.820	0.000	0.404
Education (0 to 4) *	2.50	1.48	Mann-Whitney U	5.194	0.000	0.574
Farm size (ha)	7.82	10.15	*t*-test	1.845	0.069	0.202
Membership in associations (0 to 1)	0.61	0.76	Chi-square	2.137	0.144	0.161
Episode of pesticide intoxication (0 to 1)	0.33	0.35	Chi-square	0.019	0.891	0.015
Perception of pesticide hazard (0 to 1)	0.58	0.46	Chi-square	1.30	0.254	0.126

* 0 = no education, 1 = primary education, 2 = lower secondary education, 3 = upper secondary education, 4 = tertiary education.

**Table 2:** Knowledge of pesticide use between trained and non-trained farmers.

Item	Trained (%)	Non-Trained (%)
Which type of pesticide application procedure involves the uniform application of a pesticide to an entire area or field?		
Directed-spray application	2.8	30.4
Broadcast application [C]	72.2	13.1
Band application	25.0	56.5
Chi-square = 31.269, *p* = 0.000		
Which technique would help to minimize off target drift during spraying?		
Use the largest droplets for coverage [C]	61.1	17.4
Increase the height of the nozzles	11.1	4.3
Increase pressure and travel speed	27.8	78.3
Chi-square = 20.988, *p* = 0.000		
Which statement about disposing of pesticide containers is true?		
Pour the rinsates down the drain	2.8	21.7
Disposed of in accordance with the label [C]	94.4	76.1
Reuse the containers	2.8	2.2
Chi-square = 6.251, *p* = 0.043		
Which property of a pesticide would make it more likely to move with water in surface runoff?		
High solubility [C]	44.4	26.1
High adsorption	25.0	34.8
High volatility	30.6	39.1
Chi-square = 3.047, *p* = 0.2179		
Which parts of the body are most likely to be exposed to pesticides during spraying?		
The hands and forearms [C]	72.2	34.8
The eyes and lungs	11.1	39.1
The feet and legs	16.7	26.1
Chi-square = 12.253, *p* = 0.002		

[C]: correct answer.

**Table 3:** Beliefs about pesticide hazard control between trained and non-trained farmers.

Item	Trained (%)	Non-Trained (%)
Do you think that pesticides harm your health?		
Not at all	2.8	17.4
Not enough to cause worry	5.5	43.5
Enough to cause a little worry	11.1	26.1
Enough to worry a great deal	80.6	13.0
Chi-square = 38.641, *p* = 0.000		
How well do you think the safety precautions work for personal safety from pesticides?		
Not at all	5.6	17.5
Not well	8.3	47.8
Quite well	13.9	21.7
Very well	72.2	13.0
Chi-square = 31.455, *p* = 0.000		
How much control do you feel you have over avoiding harmful effects of pesticides?		
No control	13.9	32.6
A little control	13.9	28.3
Some control	38.9	26.1
A lot of control	33.3	13.0
Chi-square = 9.633, *p* = 0.022		

**Table 4:** Safety behaviors between trained and non-trained farmers.

Item	Trained (%)	Non-Trained (%)
Do you check your spraying equipment before pesticide application?		
Always	72.2	13.0
Sometimes	25.0	56.6
Never	2.8	30.4
Chi-square = 31.2693, *p* = 0.000		
Do you wear gloves when you prepare the spraying solution?		
Always	52.8	4.3
Sometimes	30.5	43.5
Never	16.7	52.2
Chi-square = 26.347, *p* = 0.000		
Do you wash your hands after pesticide application?		
Always	91.7	76.1
Sometimes	5.5	19.6
Never	2.8	4.3
Chi-square = 3.682, *p* = 0.158		
Do you take a shower after pesticide application?		
Always	91.7	69.6
Sometimes	5.5	26.1
Never	2.8	4.3
Chi-square = 6.367, *p* = 0.041		

**Table 5:** Overall scores for farmers’ knowledge, behaviors, and beliefs.

Variable	Group	Mean	*t*-Test	*p*-Value	Effect Size
Knowledge	Trained	2.72	−5.52	0.000	0.525
	Non-trained	1.33			
Behaviors	Trained	6.83	−6.01	0.000	0.563
	Non-trained	4.72			
Beliefs	Trained	7.14	−6.29	0.000	0.575
	Non-trained	3.84			

## References

[1] Damalas C.A. (2009). Understanding benefits and risks of pesticide use. Sci. Res. Essays.

[2] Khan M., Damalas C.A. (2015). Factors preventing the adoption of alternatives to chemical pest control among Pakistani cotton farmers. Int. J. Pest Manag..

[3] Carvalho F.P. (2017). Pesticides, environment, and food safety. Food Energy Secur..

[4] Mengistie B.T., Mol A.P.J., Oosterveer P. (2017). Pesticide use practices among smallholder vegetable farmers in Ethiopian Central Rift Valley. Environ. Dev. Sustain..

[5] Damalas C.A., Khan M. (2017). Pesticide use in vegetable crops in Pakistan: Insights through an ordered probit model. Crop Prot..

[6] Baharuddin M.R.B., Sahid I.B., Noor M.A., Sulaiman N., Othman F. (2011). Pesticide risk assessment: A study on inhalation and dermal exposure to 2,4-D and paraquat among Malaysian paddy farmers. J. Environ. Sci. Health Part B.

[7] Damalas C.A., Koutroubas S.D. (2016). Farmers’ exposure to pesticides: Toxicity types and ways of prevention. Toxics.

[8] Goel A., Aggarwal P. (2007). Pesticide poisoning. Nat. Med. J. India.

[9] Mew E.J., Padmanathan P., Konradsen F., Eddleston M., Chang S., Phillips M.R., Gunnell D. (2017). The global burden of fatal self-poisoning with pesticides 2006-15: Systematic review. J. Affect. Disord..

[10] Damalas C.A., Eleftherohorinos I.G. (2011). Pesticide exposure, safety Issues, and risk assessment indicators. Int. J. Environ. Res. Public Health.

[11] Hajek A. (2004). Natural Enemies: An Introduction to Biological Control.

[12] Göen T., Schmidt L., Lichtensteiger W., Schlumpf M. (2017). Efficiency control of dietary pesticide intake reduction by human biomonitoring. Int. J. Hyg. Environ. Health.

[13] Macfarlane E., Chapman A., Benke G., Meaklim J., Sim M., McNeil J. (2008). Training and other predictors of personal protective equipment use in Australian grain farmers using pesticides. Occup. Environ. Med..

[14] Cohen A., Colligan M.J., Sinclair R., Newman J., Schuler R. (1998). Assessing Occupational Safety and Health Training.

[15] Jørs E., Lander F., Huici O., Morant R.C., Gulis G., Konradsen F. (2014). Do Bolivian small holder farmers improve and retain knowledge to reduce occupational pesticide poisonings after training on Integrated Pest Management?. Environ. Health.

[16] Hashemi S.M., Hosseini S.M., Damalas C.A. (2009). Farmers’ competence and training needs on pest management practices: Participation in extension workshops. Crop Prot..

[17] Baxter L.A., Babbie E.R. (2003). The Basics of Communication Research.

[18] Black K. (2011). Business Statistics for Contemporary Decision Making.

[19] Damalas C.A., Abdollahzadeh G. (2016). Farmers’ use of personal protective equipment during handling of plant protection products: Determinants of implementation. Sci. Total Environ..

[20] Marsden P.V., Wright J.D. (2010). Handbook of Survey Research.

[21] Rosenbloom T., Shahar A., Elharar A., Danino O. (2008). Risk perception of driving as a function of advanced training aimed at recognizing and handling risks in demanding driving situations. Accid. Anal. Prev..

[22] Saeed M.F., Shaheen M., Ahmad I., Zakir A., Nadeem M., Chishti A.A., Shahid M., Bakhsh K., Damalas C.A. (2017). Pesticide exposure in the local community of Vehari District in Pakistan: An assessment of knowledge and residues in human blood. Sci. Total Environ..

[23] Jallow M.F.A., Awadh D.G., Albaho M.S., Devi V.Y., Thomas B.M. (2017). Pesticide knowledge and safety practices among farm workers in Kuwait: Results of a survey. Int. J. Environ. Res. Public Health.

[24] Da Silva M., Stadlinger N., Mmochi A.J., Stålsby Lundborg C., Marrone G. (2016). Pesticide use and self-reported health symptoms among rice farmers in Zanzibar. J. Agromed..

[25] Levesque D.L., Arif A.A., Shen J. (2012). Effectiveness of pesticide safety training and knowledge about pesticide exposure among hispanic farmworkers. J. Occup. Environ. Med..

[26] Shea T., De Cieri H., Donohue R., Cooper B., Sheehan C. (2016). Leading indicators of occupational health and safety: An employee and workplace level validation study. Saf. Sci..

[27] Sacchettini G., Calliera M. (2017). Link practical-oriented research and education: New training tools for a sustainable use of plant protection products. Sci. Total Environ..

[28] Calliera M., Berta F., Galassi T., Mazzini F., Rossi R., Bassi R., Meriggi P., Bernard A., Marchis A., Di Guardo A., Capri E. (2013). Enhance knowledge on sustainable use of plant protection products within the framework of the Sustainable Use Directive. Pest Manag. Sci..

[29] Sacchettini G., Calliera M., Marchis A., Lamastra L., Capri E. (2012). The stakeholder-consultation process in developing training and awareness-raising material within the framework of the EU Directive on Sustainable Use of Pesticides: The case of the EU-project BROWSE. Sci. Total Environ..

[30] Jennings A.A., Li Z. (2014). Scope of the worldwide effort to regulate pesticide contamination in surface soils. J. Environ. Manag..

[31] Walton A.L., LePrevost C., Wong B., Linnan L., Sanchez-Birkhead A., Mooney K. (2017). Pesticides: Perceived threat and protective behaviors among Latino farmworkers. J. Agromed..

